# When recommendations meet reality: the challenge of walking in the Americas

**DOI:** 10.1016/j.lana.2026.101473

**Published:** 2026-04-10

**Authors:** The Lancet Regional Health – Americas

The benefits of an active routine are well established: a regular walk reduces the risk of chronic diseases, lowers depression, slows cognitive decline, and strengthens memory. WHO recommends 150–300 min of moderate to vigorous aerobic intensity activity per week for adults and at least 60 min per day for children and adolescents. However, Luciano and colleagues showed that even a short walk is associated with positive impacts on health. But speaking about movement remains deceptively simple in a region where even walking cannot be taken for granted.

For years, reaching 10,000 daily steps was widely promoted as a marker for health benefits. However, a 2022 meta-analysis published in *The Lancet Public Health* showed that fewer steps can already yield significant benefits, especially among older adults. A more recent study published in the same journal confirmed a dose–response pattern, indicating that benefits start well below the 10,000-step mark. A 2026 study in *eClinicalMedicine* showed that small increases in daily movement, alongside better sleep and dietary habits, are linked to increases in life expectancy. Even ordinary activities, like a 10–15-min walk to the supermarket, can contribute to meaningful health improvements. Movement, in its simplest form, remains powerful for health.

However, translating these individual benefits into everyday practice is far from straightforward in many parts of the Americas, even in high-income settings such as the USA. The ability to translate recommendations, such as walking, accumulating minutes of moderate activity, or simply moving throughout the day, into practice is largely shaped by deeply unequal social, environmental, and structural conditions.

In numerous cities across the American continent even basic infrastructure such as continuous, safe sidewalks is still an exception. Around 10% of buildings near central areas in Lima and Mexico City are inaccessible on foot due to inadequate infrastructure, like interruptions, physical blockages, and missing connections in the sidewalk network. While comparatively better conditions were seen in Buenos Aires, the proportion of continuous and accessible sidewalks remains as low as 20%. Urban violence further constrains mobility: unsafe streets limit schedules, routes, and the sense of security necessary for a walk, pushing many toward car-dependent routines.

At the same time, Latin America has generated some of the most influential initiatives aimed at making walking possible and socially acceptable in urban settings. Open Streets programmes such as the Ciclovía temporarily reallocate street space to pedestrians, creating safe conditions for walking and leisure activities in a free, public area that is accessible across age and income groups. Several community-based initiatives in Brazil and Colombia have reduced social and environmental barriers to everyday walking and evidence from these experiences shows that walking can increase when cities create conditions that supports it.

Initiatives can expand opportunities for walking, but these gains are not experienced equally. This is particularly impactful for women, who face greater risks of harassment and violence in public spaces and unequal access to income. The combination of unsafe streets and low income can leave women more dependent on others, restricting their autonomy, leisure, and active mobility. These limitations intersect with inequitable access to green infrastructure. A study across 371 cities in 11 countries in Latin America showed significant deficits and disparities in parks and green areas, driven by socioeconomic patterns and the uncontrolled expansion of urban spaces. Without safe, accessible public places and adequate infrastructure, the idea of “simply walking more” becomes unrealistic and is not very motivating for millions.

The growing digitalisation of daily life adds yet another complication. With more than five billion smartphone users worldwide, tasks that once required small bouts of movement, like paying bills, shopping, and scheduling services are now completed on screens, reducing non-exercise activity throughout the day. Digital convenience has reshaped routines in ways that often amplify sedentary behaviours, especially in dense urban settings where opportunities for movement are already scarce.

The challenge in the Americas lies not only in encouraging movement but in making walking feasible in the first place by reducing violence, ensuring accessible sidewalks, expanding green areas, and recognising active mobility as a structural component of public health. If cities continue to be built in ways that constrain mobility, the benefits of walking will remain unevenly distributed, remaining most elusive to those with fewer resources and less time to move. Global physical activity goals will only become meaningful when they confront the inequalities that determine who is able to walk in everyday life across the region.

